# Expanded Phylogenetic Diversity and Metabolic Flexibility of Mercury-Methylating Microorganisms

**DOI:** 10.1128/mSystems.00299-20

**Published:** 2020-08-18

**Authors:** Elizabeth A. McDaniel, Benjamin D. Peterson, Sarah L. R. Stevens, Patricia Q. Tran, Karthik Anantharaman, Katherine D. McMahon

**Affiliations:** a Department of Bacteriology, University of Wisconsin—Madison, Madison, Wisconsin, USA; b Department of Integrative Biology, University of Wisconsin—Madison, Madison, Wisconsin, USA; c Wisconsin Institute for Discovery, University of Wisconsin—Madison, Madison, Wisconsin, USA; d American Family Insurance Data Science Institute, University of Wisconsin—Madison, Madison, Wisconsin, USA; e Department of Civil and Environmental Engineering, University of Wisconsin—Madison, Madison, Wisconsin, USA; f Environmental Chemistry and Technology Program, University of Wisconsin—Madison, Madison, Wisconsin, USA; University of Illinois at Urbana—Champaign

**Keywords:** genomics, mercury, metagenomics, methylmercury

## Abstract

Methylmercury is a potent bioaccumulating neurotoxin that is produced by specific microorganisms that methylate inorganic mercury. Methylmercury production in diverse anaerobic bacteria and archaea was recently linked to the *hgcAB* genes. However, the full phylogenetic and metabolic diversity of mercury-methylating microorganisms has not been fully unraveled due to the limited number of cultured experimentally verified methylators and the limitations of primer-based molecular methods. Here, we describe the phylogenetic diversity and metabolic flexibility of putative mercury-methylating microorganisms by *hgcAB* identification in publicly available isolate genomes and metagenome-assembled genomes (MAGs) as well as novel freshwater MAGs. We demonstrate that putative mercury methylators are much more phylogenetically diverse than previously known and that *hgcAB* distribution among genomes is most likely due to several independent horizontal gene transfer events. The microorganisms we identified possess diverse metabolic capabilities spanning carbon fixation, sulfate reduction, nitrogen fixation, and metal resistance pathways. We identified 111 putative mercury methylators in a set of previously published permafrost metatranscriptomes and demonstrated that different methylating taxa may contribute to *hgcA* expression at different depths. Overall, we provide a framework for illuminating the microbial basis of mercury methylation using genome-resolved metagenomics and metatranscriptomics to identify putative methylators based upon *hgcAB* presence and describe their putative functions in the environment.

**IMPORTANCE** Accurately assessing the production of bioaccumulative neurotoxic methylmercury by characterizing the phylogenetic diversity, metabolic functions, and activity of methylators in the environment is crucial for understanding constraints on the mercury cycle. Much of our understanding of methylmercury production is based on cultured anaerobic microorganisms within the Deltaproteobacteria, *Firmicutes*, and *Euryarchaeota.* Advances in next-generation sequencing technologies have enabled large-scale cultivation-independent surveys of diverse and poorly characterized microorganisms from numerous ecosystems. We used genome-resolved metagenomics and metatranscriptomics to highlight the vast phylogenetic and metabolic diversity of putative mercury methylators and their depth-discrete activities in thawing permafrost. This work underscores the importance of using genome-resolved metagenomics to survey specific putative methylating populations of a given mercury-impacted ecosystem.

## Abstract (precis)

Accurately assessing the production of bioaccumulative neurotoxic methylmercury by characterizing the phylogenetic diversity, metabolic functions, and activity of methylators in the environment is crucial for understanding constraints on the mercury cycle. Much of our understanding of methylmercury production is based on cultured anaerobic microorganisms within the Deltaproteobacteria, *Firmicutes*, and *Euryarchaeota.* Advances in next-generation sequencing technologies have enabled large-scale cultivation-independent surveys of diverse and poorly characterized microorganisms from numerous ecosystems. We used genome-resolved metagenomics and metatranscriptomics to highlight the vast phylogenetic and metabolic diversity of putative mercury methylators and their depth-discrete activities in thawing permafrost. This work underscores the importance of using genome-resolved metagenomics to survey specific putative methylating populations of a given mercury-impacted ecosystem.

## INTRODUCTION

Methylmercury (MeHg) is a potent neurotoxin that biomagnifies upward through food webs. Inorganic mercury deposited in sediments, freshwater, and the global oceans from both natural and anthropogenic sources is converted to bioavailable and biomagnifying organic MeHg (1, 2). The majority of MeHg exposure stems from contaminated marine seafood consumption (3), which is the most problematic for childbearing women and results in severe neuropsychological deficits when developing fetuses are exposed (4, 5). MeHg production in the water column and sediments of freshwater lakes is one of the leading environmental sources that causes fish consumption advisories (6, 7). Biotransformation from inorganic mercury to organic methylmercury has been thought to be carried out by specific anaerobic microorganisms, namely, sulfur-reducing (SRB) and iron-reducing (FeRB) bacteria and methanogenic archaea (8–11). Early identification of mercury-methylating microorganisms relied on testing isolates cultured from anaerobic sediments for MeHg production after spiking with Hg (11, 12). Associations of microbial community structure with Hg speciation and biogeochemical characteristics in the environment were inferred through profiling of the 16S rRNA gene (13, 14). However, mercury methylation potential cannot be predicted based on 16S rRNA phylogenetic signal and is, rather, a species- or strain-specific trait (10, 15–18).

Recently, the *hgcAB* genes were discovered to be required for mercury methylation in several model organisms (19). The *hgcA* gene encodes a corrinoid-dependent enzyme that is predicted to act as a methyltransferase, and *hgcB* encodes a 2[4Fe-4S] ferredoxin that reduces the corrinoid cofactor (19). Importantly, both genes are thus far known to only occur in microorganisms capable of methylating mercury and are both required for methylation activity. This discovery has allowed for high-throughput identification of microorganisms with methylation potential in cultures or diverse environmental data sets based upon *hgcAB* sequence presence (17, 20). Clone and amplicon sequencing has been used as a low-cost method to retrieve *hgcAB* sequences with sufficient sequencing depth to assess the abundance and diversity of the *hgcAB* gene pair in the environment (18, 21–26). Broad-range and clade-specific quantitative PCR (qPCR) primers have been developed to screen for and quantify specific methylating groups in a given site (18, 21, 22). Identification of *hgcAB* sequences on metagenomic contigs has expanded the phylogenetic diversity of putative methylators beyond canonical SRBs, FRBs, and methanogens to include members of the *Aminicenantes*, *Chloroflexi*, *Elusimicrobia*, *Nitrospina*, *Nitrospirae*, *Spirochaetes*, and other groups (18, 20, 27, 28). However, *hgcAB* gene sequencing approaches have been shown to fail to capture the true phylogenetic diversity of putative methylating microorganisms (29), and the metabolic capabilities of identified microorganisms cannot be reliably inferred using these techniques.

Genome-resolved metagenomics is a powerful approach to connect the phylogenetic identity of microorganisms with metabolic potential through reconstructed population genomes from the environment (30). These genomes can then be used in conjunction with metatranscriptomics and metaproteomics to further explore the functional potential, activity, and putative interactions of the microbial community (31). Jones et al. reconstructed metagenome-assembled genomes from two sulfate-impacted lakes in which the dominant putative methylators consisted of members of the *Spirochaetes*, *Aminicenantes*, and PVC superphylum (29). Gionfriddo et al. applied assembly-based and genome-resolved metagenomics to understand microbial mercury resistance and mercury methylation processes in geothermal springs (32). We recently investigated methylation potential across the anoxic food web of Lake Mendota and recovered population genomes of putative methylators among the *Bacteroidetes* and PVC superphylum with fermentation capabilities (33).

In this study, we identified nearly 1,000 genomes containing the *hgcA* marker from publicly available isolate genomes, metagenome-assembled genomes (MAGs), and novel bins assembled from three freshwater lakes. Using this collection of MAGs and reference genomes, we identified putative methylators spanning 30 phyla from diverse ecosystems, including phyla that, to our knowledge, have never been characterized as methylating groups. The *hgcAB* phylogenetic signal further confirms that this diverse locus originated through extensive horizontal gene transfer events, and provides insights into the difficulty of predicting the identity of putative methylators using ribosomal sequences or broad-range *hgcA* primers (16, 20, 29). Putative methylators span metabolic guilds beyond canonical SRBs, FRBs, and methanogenic archaea, carrying genes for traits such as nitrogen fixation and metal resistance pathways. To understand *hgcA* expression in the environment, we analyzed depth-discrete metatranscriptomes from a permafrost thawing gradient from which we identified 111 putative methylators. This work demonstrates the significance of reconstructing population genomes from the environment to accurately capture the phylogenetic distribution and metabolic capabilities of putative methylating groups in a given system.

## RESULTS AND DISCUSSION

### Identification of diverse, novel, putative mercury-methylating microorganisms.

**FIG 1:**
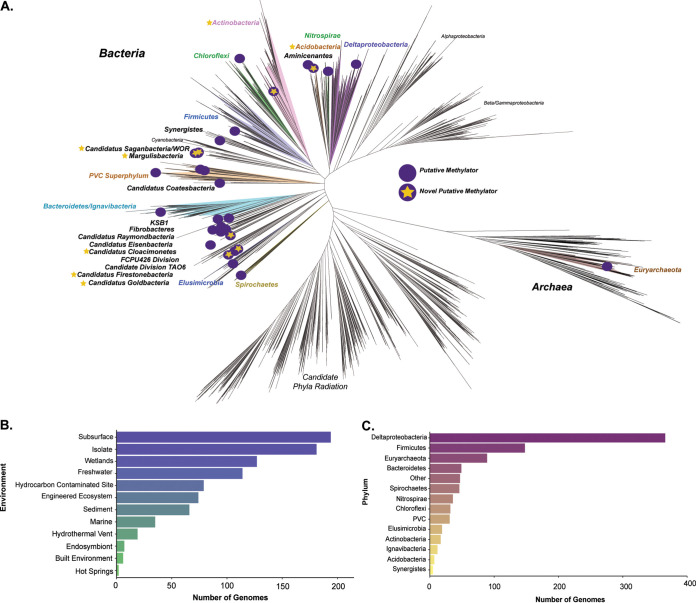
Phylogenetic distribution and diversity of putative methylators. (A) Putative methylators among the prokaryotic tree of life. The highest quality putative methylator from each identified phylum was selected as a representative among select references from Anantharaman et al. (39) from across the tree of life. The tree was constructed from an alignment of 16 concatenated ribosomal proteins. Purple dots represent putative methylating phyla, and those with stars represent phyla containing putative novel methylating lineages that, to our knowledge, have not been identified as potential mercury methylators before this study. Groups in bold and/or colored are putative methylating groups, whereas a few uncolored groups in nonboldface font are noted only for orientation. (B) Numbers of putative methylating genomes found in various environments or genomes sequenced from isolates. (C) Numbers of putative methylating genomes belonging to each phylum. Phylum names are given for all groups except the Deltaproteobacteria class of *Proteobacteria*, as no putative *Proteobacteria* methylators were identified outside the Deltaproteobacteria. The group “other” contains several candidate phyla and genomes within groups with few representatives, described in Fig. S1 in the supplemental material.

We identified nearly 1,000 putative bacterial and archaeal mercury methylators spanning 30 phyla among publicly available isolate genomes and MAGs recovered from numerous environments (Fig. 1). Well-known methylators among the Deltaproteobacteria, *Firmicutes*, and *Euryarchaeota* represent well more than one-half of all identified putative methylators, with the majority among the Deltaproteobacteria (Fig. 1C). We also expanded upon groups of methylators that, until recently, have not been considered to be major contributors to mercury methylation. Putative methylators belonging to the *Spirochaetes* and the PVC superphylum (consisting of *Planctomycetes*, *Verrucomicrobia*, *Chlamydiae*, and *Lentisphaerae* phyla and several candidate divisions) were previously identified from metagenomic contigs (18) and reconstructed MAGs from Jones et al. (29). Additionally, *hgcA* sequences have been detected on metagenomic contigs belonging to the *Nitrospirae*, *Chloroflexi*, and *Elusimicrobia* (18, 27), but overall, it is unknown how these groups contribute to MeHg production. We also detected *hgcA* in phyla that, to our knowledge, have not been shown to carry the gene. We identified 7 *hgcA*^+^ *Acidobacteria*, 5 of which come from the permafrost gradient system, where they are considered to be the main plant biomass degraders (31). We also identified 17 *hgcA*^+^ *Actinobacteria*, which all belong to poorly characterized lineages within the *Coriobacteriia* and *Thermoleophilia* classes, as described below. A few putative methylators belong to recently described candidate phyla, such as “*Candidatus* Aminicenantes,” “*Candidatus* Firestonebacteria,” and WOR groups (see Fig. S1D in the supplemental material).

10.1128/mSystems.00299-20.1 FIG S1 Overall putative methylating genome and MAG statistics. All summaries and statistics represented in this figure and additional metadata are available in Table S1 available at https://figshare.com/articles/dataset/mehg-metadata/10062413. (A) Numbers of putative methylators that are isolates and the phylum to which they belong. (B) Estimated genome sizes of all putative methylating isolates. (C) Estimated GC contents of all putative methylating isolates. (D) Estimated genome sizes of all MAGs not in the “other” group. (E) Estimated GC contents of all MAGs not in the “other” group. (F) Genome quality statistics of estimated completeness and contamination as calculated by CheckM for each MAG. Only medium quality (>50% complete and <10% redundancy) putative methylating MAGs were included in the dataset. (G) Summary of MAGs that are categorized as “other” in Fig. 1C and the phylum they belong to. (H) Estimated genome sizes and GC contents for all genomes in the “other” category. Download FIG S1, TIF file, 0.5 MB. Copyright © 2020 McDaniel et al. 2020 McDaniel et al. This content is distributed under the terms of the Creative Commons Attribution 4.0 International license.

We recently reconstructed new MAGs from three freshwater lakes with diverse biogeochemical characteristics. Trout Bog Lake is a small, dimictic humic lake in a rural area near Minocqua, WI, that is surrounded by sphagnum moss, which leaches large amounts of organic carbon. Lake Tanganyika is a meromictic lake with an anoxic monimolimnion in the East African Rift Valley and is the second largest lake in the world both by volume and depth, from which we recently reconstructed nearly 4,000 MAGs (34). Lake Mendota is a large, dimictic eutrophic lake in an urban setting in Madison, WI, with a sulfidic anoxic hypolimnion. From these reconstructed freshwater MAGs, we identified 55 putative mercury methylators. Notably, several understudied PVC clades were recovered from the Lake Mendota hypolimnion. Members of this superphylum are ubiquitous in freshwater and marine environments, display a cosmopolitan distribution, and can make up approximately 7% of the total microbial community in these ecosystems (35–38). In Lake Mendota, members of the PVC superphylum account for approximately 40% of the microbial community in the hypolimnion, the majority of which do not contain *hgcA* (33).

A majority of the identified methylators were from a subsurface aquifer system and thawing permafrost gradient assembled by Anantharaman et al. (39) and Woodcroft et al. (31), respectively (Fig. 1B). Additionally, we identified methylators from hydrocarbon-contaminated sites such as oil tills and sand ponds, sediments and wetlands, engineered systems, hydrothermal vents, and the built environment. Only a few *hgcA*^+^ organisms were recovered from marine systems, as the Tara Ocean project mostly includes samples from surface waters (40) and therefore would not include traditionally anaerobic methylating microorganisms. Although not as numerous as MAGs from various metagenomic surveys, we identified *hgcA* in more than 150 isolate genomes, with some among the *Bacteroidetes*, *Spirochaetes*, *Nitrospirae*, and *Chloroflexi* (Fig. S1A). Using an extensive set of genomes mostly recovered from anoxic environments, we were able to expand upon the known phylogenetic diversity of putative mercury-methylating microorganisms.

### Novel *Actinobacteria* lineages with putative methylating potential.

To our knowledge, members of the *Actinobacteria* have never been characterized as putative methylators or found to contain the *hgcA* marker. We identified 17 *hgcA*^+^ *Actinobacteria* (16 of which also contained *hgcB*) among diverse environments, such as our Trout Bog and Lake Mendota freshwater data sets, as well as within the permafrost gradient and MAGs recovered from hydrocarbon contaminated sites (31, 41). In freshwater systems, *Actinobacteria* are ubiquitous and present a cosmopolitan distribution, with specific lineages comprising up to 50% of the total microbial community (42, 43). Freshwater *Actinobacteria* traditionally fall within the class *Actinobacteria* but have a lower abundance distribution in the hypolimnion due to decreasing oxygen concentrations (44, 45). However, none of the *hgcA*^+^ *Actinobacteria* belong to ubiquitous freshwater lineages.

All of the 17 identified *hgcA*^+^ *Actinobacteria* belong to poorly described classes or ill-defined lineages that may branch outside the established six classes of *Actinobacteria* (46). Nine fall within the *Coriobacteriia* class, four fall within the *Thermoleophilia* class, and the other four diverge from named *Actinobacteria* lineages. Three of the ill-defined *Actinobacteria* MAGs have been designated within the proposed class UBA1414, with two assembled from the permafrost system and one from the Lake Mendota hypolimnion. The other ill-defined *Actinobacteria* MAG was designated within the proposed class RBG-13-55-18 and was also recovered from the permafrost system. While some *Coriobacteriia* have been identified as clinically significant members of the human gut, all identified putative methylators belong to the OPB41 order (47), which refers to the 16S rRNA sequence identified by Hugenholtz et al. from the Obsidian Pool hot spring in Yellowstone National Park (48). Members of the OPB41 order have also been found to subsist along the subseafloor of the Baltic Sea, an extreme and nutrient-poor environment (49).

**FIG 2:**
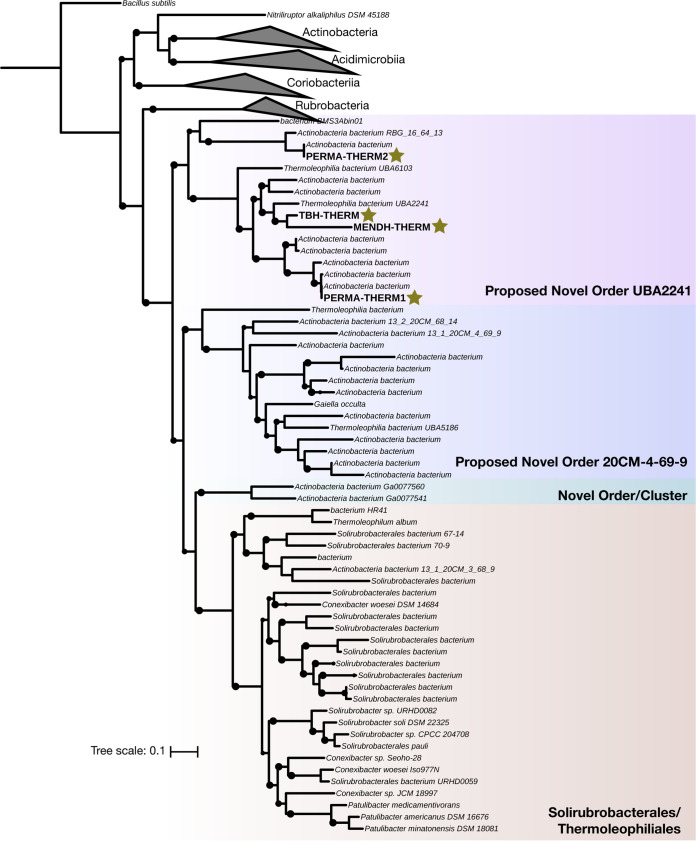
Novel putative methylators within the *Thermoleophilia* class of *Actinobacteria.* Phylogenetic tree of publicly available and assembled *Thermoleophilia* MAGs and *Actinobacteria* references. Representative or reference genomes belonging to the other 5 classes of *Actinobacteria* were used, with Bacillus subtilis used as an outgroup. Orders are clustered and colored by GTDB-proposed designation. The phylogeny was constructed with RAxML using 100 rapid bootstraps, and nodes with bootstrap support greater than 60 are shown as black circles. Stars denote putative methylators identified in this study.

Members of the *Thermoleophilia* are known as heat- and oil-loving microbes due to their growth restriction to only substrate *n*-alkanes (50). Within this deep-branching lineage, the two orders *Solirubrobacterales* and *Thermoleophilales* are currently recognized based on sequenced isolates and the most updated version of Bergey’s taxonomy (46, 51). However, our *hgcA^+^ Thermoleophilia* MAGs from the hypolimnia of Lake Mendota and Trout Bog as well as two other *hgcA^+^ Thermoleophilia* from the permafrost thawing gradient do not cluster within the recognized orders (Fig. 2). According to the genome taxonomy database (GTDB), these MAGs cluster within a novel order preliminarily named UBA2241, as the only MAGs recovered from this novel order to date have been from the authors’ corresponding permafrost system (31, 52).

Based on collected isolates, members of the *Thermoleophilia* are assumed to be obligately aerobic (51). However, the functional potential of *Thermoleophilia* members has been poorly described due to few sequenced isolates and high-quality MAGs (53). We reconstructed the main metabolic pathways of a high-quality *Thermoleophilia* MAG, referred to as MENDH-Thermo (see Table S2 available at https://figshare.com/articles/dataset/MENDH-Thermo-annotations/11620104). All components of the glycolytic pathway are present for using a variety of carbohydrates for growth. A partial tricarboxylic acid (TCA) cycle for providing reducing power is present; only missing the step for converting citrate to isocitrate, which can be transported from outside sources. Interestingly, the MENDH-Thermo genome encodes the tetrahydromethanopterin-dependent enzymes encoded by *mch* and *fwdAB*, pointing to either formate utilization or detoxification (54, 55). We could not detect the presence of a putative acetate kinase; therefore, autotrophic growth through the formation of acetate is unlikely. Interestingly, the MENDH-Thermo genome encodes a full pathway for the formation of 4-hydroxybutryate, a storage polymer (56). As the MENDH-Thermo genome can also synthesize the amino acids glycine and serine, combined with the ability to use reduced carbon compounds and form storage polymers, the MENDH-Thermo genome could exhibit a methylotrophic lifestyle but is missing key steps for tetrahydromethanopterin cofactor synthesis and formate oxidation (57). The identification of putative methylators within the *Thermoleophilia* highlights novel metabolic features not only of putative methylators but also of a poorly described class within *Actinobacteria*.

### Implications for horizontal gene transfer of *hgcAB*.

**FIG 3:**
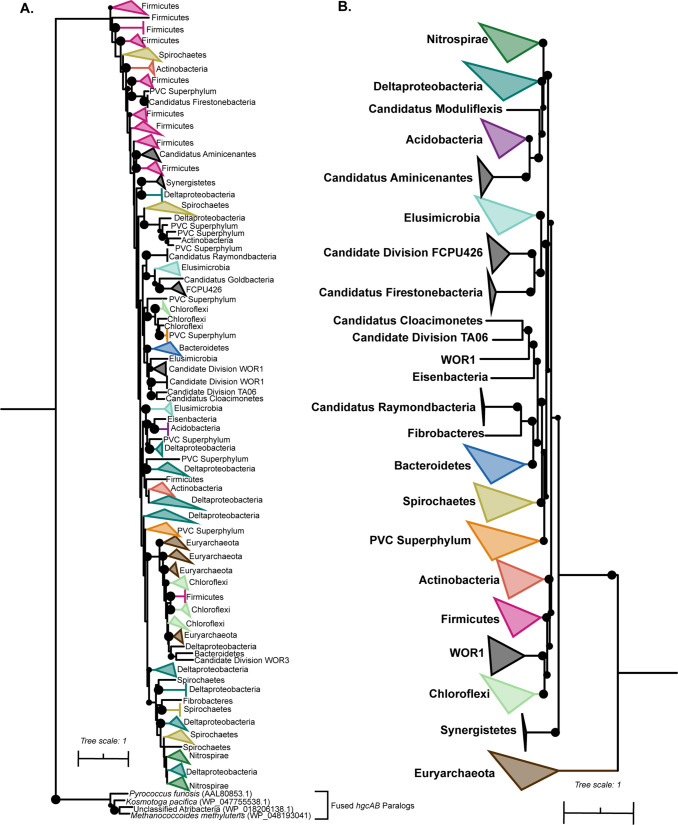
Discordance of HgcAB phylogeny relative to a concatenated corresponding ribosomal protein phylogeny of select methylators. (A) Phylogeny of HgcAB of select methylators was constructed as described in Materials and Methods. The tree was constructed using RAxML with 100 rapid bootstraps, and nodes with bootstrap support greater than 50 are depicted as black circles. (B) Corresponding concatenated ribosomal protein phylogeny of the select methylators. The tree was constructed using a set of 16 ribosomal proteins (2,470 positions following masking), concatenated, and built using RAxML with 100 rapid bootstraps. Nodes with bootstrap support of greater than 60 are denoted as black circles. A fully annotated HgcAB tree is provided in Fig. S4 at https://figshare.com/articles/figure/hgcAB_annotated_tree_full/12592085.

The HgcAB protein phylogeny does not follow a logical species tree phylogeny, as demonstrated by a comparison to a concatenated phylogeny of ribosomal proteins (Fig. 3). For example, *Deltaproteobacteria* HgcAB sequences are some of the most disparate sequences surveyed, clustering with HgcAB sequences of *Actinobacteria*, *Nitrospirae*, *Spirochaetes*, and members of the PVC superphylum. Although *Firmicutes* HgcAB sequences are not as disparate, they also cluster with HgcAB sequences of other phyla such as Deltaproteobacteria and *Spirochaetes.* The only HgcAB sequences that clustered monophyletically among our data set are the *Bacteroidetes/Ignavibacteria* groups and *Nitrospirae*. These observations have implications for the underlying evolutionary mechanisms of the methylation pathway and modern methods for identifying methylating populations in the environment.

Gene gain/loss mediated through several horizontal gene transfer (HGT) events was previously suggested to underpin the sparse and divergent phylogenetic distribution of *hgcAB* (17, 19–21, 29). Metabolic functions can be horizontally transferred across diverse phyla through numerous mechanisms, such as phage transduction, transposon-mediated insertion of genomic islands or plasmid exchanges, direct conjugation, and/or gene gain/loss events. We were unable to identify any *hgcA*-like sequences on any viral contigs in the NCBI database or within close proximity to any known insertion sequences or transposases, and all known *hgcA* sequences have been found on the chromosome. Structural and mechanistic similarities between the cobalamin-binding domains of HgcA and the carbon monoxide dehydrogenase/acetyl coenzyme A (acetyl-CoA) synthase (CODH/ACS) of the reductive acetyl-CoA pathway (also known as the Wood-Ljungdahl pathway) suggests associations between the two pathways (19, 20). Interestingly, *hgcA* sequences of *Euryarchaeota* and *Chloroflexi* cluster together, which was recently found to also be true for the CODH of these groups (58). It is plausible that a gene duplication event followed by rampant gene gain/loss and independent transfers of both the CODH and HgcA proteins, respectively, could explain the disparate phylogeny, as has been described for both pathways (19, 20, 58).

**FIG 4:**
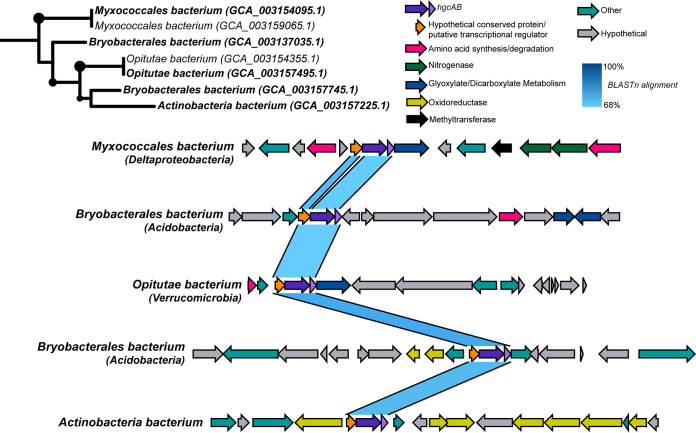
Gene neighborhoods of diverse permafrost methylators containing similar *hgcAB* regions. Contigs containing similar *hgcAB* regions from putative methylators identified in a permafrost thawing system (31) were compared. Pairwise nucleotide BLAST between the genes for each contig was performed and visualized in Easyfig (106). Colors correspond to predicted functions of each gene. *hgcAB* genes were identified based on curated HMMs and reference sequences, while all other gene assignments were made based on Prokka predictions.

To illustrate an example of potential interphylum HGT of *hgcAB*, we selected methylators assembled from a permafrost system containing *hgcAB* regions with approximately 70% DNA sequence similarity (Fig. 4). Five putative methylators encompassing four different phyla (one Deltaproteobacteria, two *Acidobacteria*, one *Verrucomicrobia*, and an *Actinobacteria*) contain highly similar *hgcAB* regions, and their *hgcAB* phylogeny does not match the species tree. All *hgcAB* sequences of these methylators are preceded by a similar hypothetical protein that is annotated as a putative transcriptional regulator in the *Opitutae* genome. We screened for this putative transcriptional regulator among all methylators in our data set and identified it immediately upstream of *hgcAB* in 112 genomes (see Table S3 available at https://figshare.com/articles/dataset/hgcAB-regulator-resultsUntitled_Item/11620107). Overall, the gene neighborhoods of these similar permafrost *hgcAB* sequences do not share any other obvious sequence or functional similarities. The *hgcAB* genes are flanked by numerous hypothetical proteins, various amino acid synthesis and degradation genes, and oxidoreductases for core metabolic pathways. More detailed phylogenomic analyses will need to be applied in future studies to specifically pinpoint individual *hgcAB* HGT events and mechanisms.

Broad-range and clade-specific qPCR primers targeting *hgcAB* have been used to screen for putative methylators in the environment (18, 22). However, these primers were constructed based on a few experimentally verified methylators, which surely does not capture the complete or true diversity of methylating microorganisms. We tested the accuracy of these primers *in silico* on the coding regions of *hgcA* in all our identified putative methylators by using the taxonomical assignment of each putative methylator from the full genome classification. Depending on the number of *in silico* mismatches allowed, only experimentally verified methylators could be identified, or *hgcA* sequences belonging to other phyla would be hit (see Table S4 at https://figshare.com/articles/dataset/hgcA-in-silico-primer-stats/10093535). For example, Deltaproteobacteria*-*specific *hgcA* qPCR primers picked up related *Actinobacteria*, *Spirochaetes*, and *Nitrospirae* sequences. Whereas the *Firmicutes*-specific primers detected all experimentally verified *Firmicutes* methylators and a few environmental *hgcA* sequences, overall, they missed the diversity of *Firmicutes hgcA* sequences.

Discordance between the HgcAB and ribosomal protein phylogenies suggests that nonspecific amplicon approaches and broad-range qPCR assays will not yield easily interpretable results. Although new broad-range *hgcAB* primers have been developed based on an updated reference database of *hgcAB* sequences (21), the apparent HGT dynamics of this gene pair makes assigning the taxonomy of resulting hits challenging. Instead, finer-scale primers of specific methylating groups could be constructed based on *hgcAB* sequences from assembled population genomes from specific environments. However, this approach relies on sufficient depth of sequencing coverage to adequately assemble *hgcAB*-containing MAGs, which may consist of low-abundance members of the community. For example, we were able to assemble genomes of 35 putative methylators out of 228 total nonredundant genomes from Lake Mendota. Anantharaman et al. (39) and Woodcroft et al. (31) generated genome-resolved metagenomic data sets at terabase scales, from which we were able to identify 111 putative methylators from a total of 2,540 genomes and 111 putative methylators from a total of 1,529 total genomes, respectively. Alternatively, methods such as multiplexed digital droplet PCR (ddPCR) approaches could be applied to simultaneously amplify the 16S rRNA and *hgcAB* genes (59, 60). This approach would allow for higher sequencing depth at a lower cost than metagenomics while also ensuring that methylation status is linked to phylogenetic identity with fidelity.

### Metabolic capabilities of identified putative methylators.

**FIG 5:**
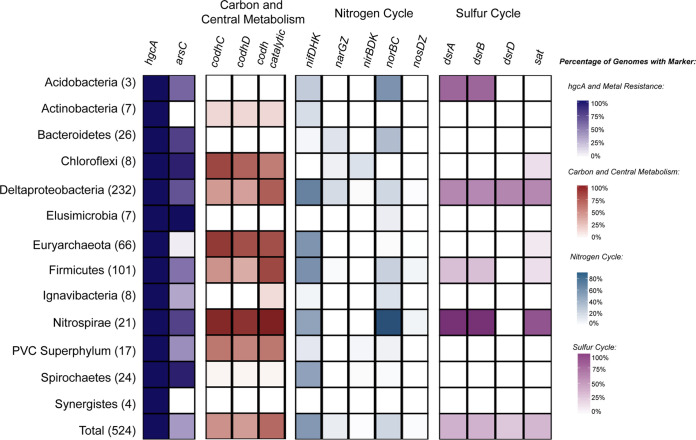
Summary of broad metabolic characteristics present among high-quality putative methylators. Genomes with >90% completeness and <10% redundancy and belonging to phyla with more than 5 genomes in that group were screened. HMM profiles spanning the sulfur, nitrogen, and carbon cycles and metal resistance markers were searched for. The intensity of shading within a cell equates to the corresponding colored legend of percentage of genomes within that group that contain that marker. *hgcA*, gene encoding a mercury methylation corrinoid protein; *arsC*, gene encoding arsenite reductase; *codhCD/*catalytic, genes encoding carbon monoxide dehydrogenase C and D and catalytic subunits; *nifDHK*, genes encoding nitrogenase subunits; *narGZ*, genes encoding nitrate reductase subunits; *nirBDK*, genes encoding nitrite reductase subunits; *norBD*, genes encoding nitric oxide reductase subunits; *nosDZ*, genes encoding nitrous oxide reductase subunits; *dsrABD*, genes encoding dissimilatory sulfite reductase; *sat*, sulfate adenylyltransferase. Individual *nif*, *nar*, *nir*, *nor*, and *nos* subunit presence/absence results were averaged together.

We next characterized the broad metabolic capabilities among 524 highly complete putative methylators. From our analysis of metabolic profiles spanning several biogeochemical cycles (39), the *hgcAB* gene pair is the only genetic marker linking high-quality methylating genomes other than universally conserved markers (Fig. 5). We detected putative methylators with methanogenesis and sulfate reduction pathways, as is expected for archaeal and bacterial methylating guilds, respectively (11, 16, 29). Although sulfate availability has been linked to methylating bacteria and active methylation, very few guilds we examined exhibited dissimilatory sulfate reduction pathways. We detected machinery for dissimilatory sulfate reduction within the *Acidobacteria*, Deltaproteobacteria, *Firmicutes*, and *Nitrospirae*. However, for example, only one-half of the *Deltaproteobacteria* methylators screened contained the *dsrABD* subunits for sulfate reduction. Similarly, we detected few guilds with the sulfate adenylyltransferase (*sat*) marker for assimilatory sulfate reduction for incorporating sulfur into amino acids. This suggests that mercury-methylating groups may be composed of guilds other than canonical SRBs and methanogenic archaea.

We found that several methylating guilds contain the essential subunits that form the nitrogenase complex for nitrogen fixation. The three main components of the nitrogenase complex are *nifH* and *nifD-nifK*, which encode the essential iron and molybdenum-iron protein subunits, respectively. We detected machinery for the nitrogenase complex within the *Acidobacteria*, *Actinobacteria*, Deltaproteobacteria, *Euryarchaeota*, *Firmicutes*, *Nitrospirae*, and *Spirochaetes* methylators. Notably, more than one-half of all *Deltaproteobacteria* methylators and approximately one-half of all *Firmicutes* methylators contain all three subunits for the nitrogenase complex. Recently, the presence of nitrate has been suggested to regulate MeHg concentrations (61), leading to nitrate addition in an attempt to control MeHg accumulation in freshwater lakes (62). Although we detected parts of the denitrification pathway in a few lineages, none of the putative methylators contained a full denitrification pathway for fully reducing nitrate to nitrogen gas. Previous studies have identified putative methylators among *Nitrospina* nitrite oxidizers in marine systems (27, 28). Although we did not detect putative *Nitrospina* methylators (possibly due to these *hgcA* sequences being unbinned, within low-quality MAGs, or low-confidence hits), links between the nitrogen cycle and mercury methylation warrant further exploration.

As mentioned above, structural and functional similarities between HgcA and the carbon monoxide dehydrogenase/acetyl-CoA synthase (CODH/ACS) suggest associations between the two pathways. This also raises the question if methylators use the reductive acetyl-CoA pathway for autotrophic growth. We screened for the presence of the codhC and codhD subunits of the carbon monoxide dehydrogenase, which have been suggested as potential paralogous origins of HgcA (19, 20). Among high-quality putative methylators, we detected both subunits among *Actinobacteria*, *Chloroflexi*, Deltaproteobacteria, *Euryarchaeota*, *Firmicutes*, *Nitrospirae*, and members of the PVC superphylum. We also screened for the presence of the CODH catalytic subunit, which would infer acetyl-CoA synthesis. Although we detected the CODH catalytic subunit, it was not detected in all methylating lineages, such as the *Elusimicrobia*, *Synergistes*, and *Spirochaetes*. This suggests other growth strategies than autotrophic carbon fixation among a large proportion of methylators, such as fermentation pathways as suggested previously by Jones et al. (29).

**FIG 6:**
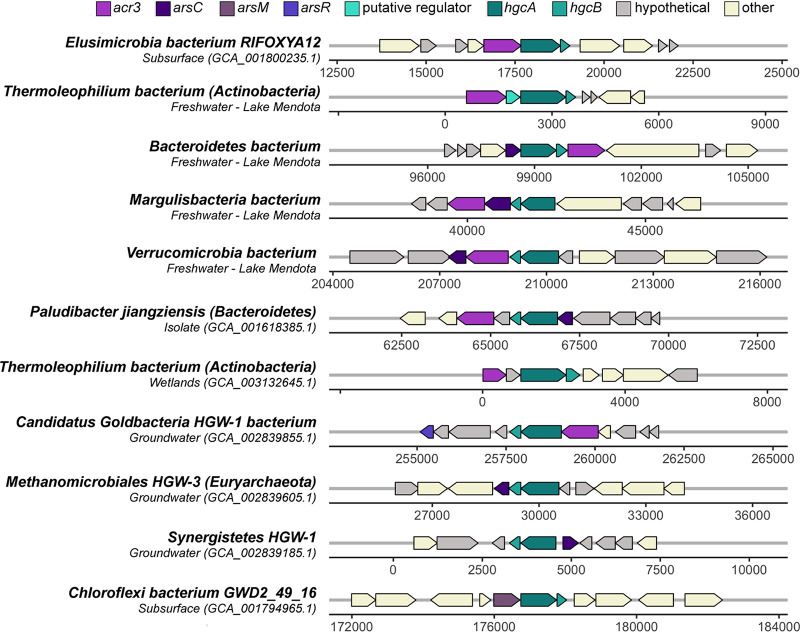
Gene neighborhoods of select putative methylators demonstrating the association of arsenic metabolism with *hgcAB* genes. Arsenic metabolism-related genes are colored shades of purple, and *hgcAB* genes and the putative regulator are colored shades of blue. *acr3*, gene encoding arsenite efflux; *arsC*, gene encoding glutaredoxin/thioredoxin-dependent arsenite reductase; *arsM*, gene encoding arsenic methyltransferase; *arsR*, gene encoding Ars-R type transcriptional regulator.

Interestingly, >50% of the putative methylators contain a thioredoxin-dependent arsenate reductase, which detoxifies arsenic through the reduction of arsenate to arsenite (63, 64). As(V) reduction to the even more toxic As(III) is tightly coupled to export from the cell, which has interesting similarities to the mercury methylation system. Hg(II) uptake has been shown to be energy dependent and therefore is potentially imported through active transport mechanisms (65). Hg(II) uptake is highly coupled to MeHg export, suggesting the methylation system may act as a detoxification mechanism against environmental Hg(II) (65). Intriguingly, numerous diverse putative methylators contain *hgcAB* regions that are immediately flanked by different arsenic-related genes or are within a few open reading frames (Fig. 6). We found several examples where the *hgcAB* region is flanked by *acr3*, encoding an arsenite efflux permease, and *arsC*, encoding a thioredoxin- or glutaredoxin-dependent arsenite reductase. Both the arsenite efflux and reductase are part of the *ars* operon for arsenic resistance and detoxification and have somewhat redundant functions in that they both achieve extrusion of toxic arsenic but through different mechanisms (66).

Recently, Goñi-Urriza et al. identified an ArsR-like regulator upstream of *hgcAB* among *Desulfovibrio* and *Pseudodesulfovibrio* methylating strains that is cotranscribed with *hgcA* (67). This family of metalloregulatory transcriptional repressors is responsive to a variety of metals such as As, Zn, and Ni, and canonical ArsR repressors mediate arsenic detoxification. (68, 69). However, some ArsR-like sequences have been identified that share homology to canonical ArsR repressors but do not contain hallmark metal binding sequence signatures (68). Recently, Gionfriddo et al. also identified a putative ArsR-like repressor downstream from *hgcAB*, in addition to ArsB, an arsenical pump membrane protein (32). We identified ArsR-like sequences in close proximity to *hgcAB* as well as *arsM*, an arsenite *S*-adenosylmethionine methyltransferase. Growing evidence for connections between metal resistance pathways and mercury methylation provide intriguing hypotheses to test concerning the constraints and controls of methylation in the environment. Overall, putative methylators are composed of metabolic guilds other than the classical SRBs and methanogenic archaea and employ diverse growth strategies other than autotrophic carbon fixation.

### Transcriptional activity of putative methylators in a permafrost system.

While the connection between the presence of the *hgcAB* genes and methylation status has been well established in cultured isolates, there have been few studies investigating *hgcAB* transcriptional activity under laboratory conditions (70) or in a specific environment (71). Additionally, the mere presence of a gene does not predict its functional dynamics or activity in the environment. Assessing microbial populations actively contributing to MeHg production is important for identifying constraints on this process, and genome-resolved metatranscriptomics may be a useful approach to do so in the environment. Woodcroft et al. performed extensive spatiotemporal sampling across three sites of the Stordalen Mire peatland in northern Sweden paired with genome-resolved metagenomics, metatranscriptomics, and metaproteomics to understand microbial contributions to carbon cycling (31). This site includes well-drained but mostly intact palsas, intermediately thawed bogs with *Sphagnum* moss, and completely thawed fens. We identified 111 *hgcA*^+^ genomes among MAGs assembled from this permafrost system spanning well-known methylators belonging to the Deltaproteobacteria, *Firmicutes*, and *Euryarchaeota* but also less-studied putative methylators within the *Acidobacteria*, *Actinobacteria*, *Elusimicrobia*, and *Verrucomicrobia*. We chose this extensive data set to explore depth-discrete activity of *hgcA*, since it is largely an anaerobic environment and methylation activity has been shown to occur in this type of system (20, 72, 73). Furthermore, there are growing concerns about MeHg production in polar regions due to thawing permafrost from global warming effects (74).

**FIG 7:**
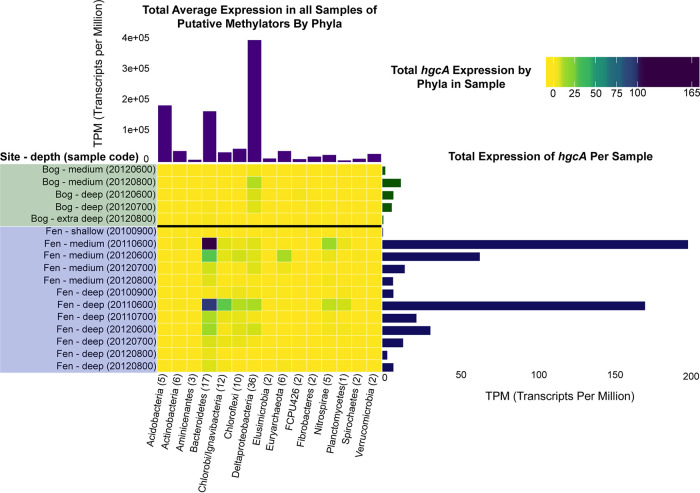
Transcriptional activity of putative methylators in a permafrost thawing gradient. Counts of mapped metatranscriptomic reads to 111 putative methylators identified in a permafrost thawing gradient in bog and fen samples (31). (Center) Total reads mapping to the *hgcA* gene within a phylum per sample normalized by transcripts per million (TPM). Heat map color scale represents a continuous gradient between 0 and 100 TPM, and a break between 100 and 165 TPM to better visualize results in the low range. (Right) Total *hgcA* counts per sample in TPM. (Top) Average overall expression of genomes within a phylum across all bog and fen samples in TPM.

We specifically investigated the site and depth-discrete expression patterns of *hgcA* within phylum-level groups (Fig. 7). We were unable to detect *hgcA* transcripts at any time point or depth among the palsa sites or the shallow bog depths; therefore, these sites and depths are not included. Lack of *hgcA* transcript detection in the frozen palsa sites may be connected to the relationship between increased methylation activity with increased amounts of oxidized organic matter (75, 76), which would more likely be present in the thawing bog and fen sites. The highest total *hgcA* transcript abundance was detected in the fen sites, which are the most thawed along the permafrost gradient. The highest *hgcA* expression was contributed by the *Bacteroidetes* in the medium and deep depths of the fen sites across multiple time points, followed by those belonging to Deltaproteobacteria in the medium and deep depths of the bog sites. Interestingly, *hgcA* expression in the bog samples was almost exclusively dominated by Deltaproteobacteria, whereas the fen samples contained a more diverse collection of phyla expressing *hgcA*, albeit *Bacteroidetes* was the most dominant. In the deep fen samples, groups such as the *Ignavibacteria*, *Chloroflexi*, and *Nitrospirae* also contributed to *hgcA* expression. Overall, methylators belonging to *Bacteroidetes* and Deltaproteobacteria also constituted the most highly transcriptionally active members across all samples.

All of the putative *Bacteroidetes* methylators transcribing *hgcA* belong to the vadinHA17 class. The name vadinHA17 was given to the 16S rRNA gene clone recovered from an anerobic digester treating winery wastewater, referring to the vinasse anerobic digestor of Narbonne (VADIN) (77). Members of the vadinHA17 group remain uncultured and are thought to be involved in hydrolyzing and degrading complex organic matter, cooccurring with methanogenic archaea (78, 79). Currently, the three publicly available *Bacteroidetes* strains containing *hgcA* belong to the *Paludibacteraceae* and *Marinilabiliaceae* and are not closely related to members of the vadinHA17 class. However, the dominant *Deltaproteobacteria* member expressing *hgcA* in the bog samples is classified within the *Syntrophobacterales* order, specifically, a member of *Smithella* spp. Among the publicly available sequenced *hgcA*^+^ *Deltaproteobacteria* isolates, 3 are among the *Syntrophobacterales* order, including the *Smithella* sp. F21 strain. This methylating strain may be an appealing experimental system to further explore *hgcA* expression patterns under different conditions.

For genomes in which we identified confident *hgcB* hits, *hgcB* expression patterns mostly mirrored that of *hgcA* (see Fig. S2A). Additionally, we identified a conserved putative regulator immediately upstream of *hgcAB* as described above that was also present in some of the permafrost MAGs. Among genomes that contained the putative regulator, we detected transcripts associated with *Chloroflexi* and Deltaproteobacteria, corresponding to sites and depths at which these groups also demonstrate similar *hgcAB* expression patterns (Fig. S2B). We also compared *hgcA* expression to that of the housekeeping gene *rpoB*, which encodes the beta subunit of RNA polymerase (see Fig. S3). There are some *hgcA*^+^ groups in which we detected appreciable *rpoB* transcripts but could not detect *hgcA* transcripts in any samples, such as the *Acidobacteria*, *Aminicenantes*, and *Fibrobacteres*. This suggests that some *hgcA*^+^ organisms are transcriptionally active but may not contribute to methylmercury production in this ecosystem. Additionally, for groups such as the *Bacteroidetes*, Deltaproteobacteria, and *Nitrospirae* in which we detected *hgcA* transcripts, we detected *rpoB* transcripts for these groups in samples in which *hgcA* was not expressed.

10.1128/mSystems.00299-20.2 FIG S2 Counts of metatranscriptomic reads to the open reading frames of *hgcB* and the conserved putative regulator upstream of *hgcAB* identified in 112 putative methylators. (A) Counts normalized in TPM mapping to *hgcB* within phylum-level groups. (B) For permafrost MAGs we identified the putative regulator in, counts normalized in TPM mapping to the putative regulator within phylum-level groups. Download FIG S2, PDF file, 0.7 MB. Copyright © 2020 McDaniel et al. 2020 McDaniel et al. This content is distributed under the terms of the Creative Commons Attribution 4.0 International license.

10.1128/mSystems.00299-20.3 FIG S3 Comparisons of expression between *hgcA* and the housekeeping gene *rpoB*. For each genome that we were able to confidently identity *rpoB* in, each point represents *hgcA* expression (*x* axis) compared to *rpoB* expression (*y* axis) across all samples and colored by the phylum that genome belongs to. (Right) The same points but split by individual samples. Download FIG S3, PDF file, 1.1 MB. Copyright © 2020 McDaniel et al. 2020 McDaniel et al. This content is distributed under the terms of the Creative Commons Attribution 4.0 International license.

Our findings have implications for understanding MeHg production in the environment, as currently, the presence of *hgcAB* is used to connect specific microorganisms to biogeochemical characteristics. However, it may be possible that only a few groups exhibit *hgcAB* activity and actually contribute to MeHg production. Although this study does not contain environmental data on inorganic mercury or MeHg concentrations, these results provide insights into *hgcA* expression for further follow-up. Previous studies of *hgcA* expression in the lab and the environment have inferred that *hgcA* is constitutively expressed (70, 71). Additionally, *hgcAB* expression by Desulfovibrio dechloracetivorans strain BerOc1 was found to have no quantifiable relationship to methylmercury production and depended upon growth conditions rather than induction of the gene pair (70). Therefore, further work is required to understand the regulation and environmental conditions leading to *hgcAB* expression and, ultimately, methylation activity. Although previous studies have inferred that *hgcAB* expression may not be connected to actual methylmercury production, genome-resolved metatranscriptomics can begin to assess these questions in the natural environment, since laboratory experiments of pure cultures may not reflect environmental conditions or dynamics. Future experiments could integrate genome-resolved metagenomics and metatranscriptomics with Hg/MeHg assays in sites with MeHg accumulation to understand if the expression of specific *hgcA*^+^ populations contributes to environmentally relevant MeHg levels. Experiments performed with concurrent collections of biogeochemical characteristics could begin to parse which environmental conditions contribute to community activity and, specifically, *hgcAB* activity. Careful replication of conditions and necessary controls could also illuminate other genes and pathways associated with the activity of the *hgcAB* genes (80). Although it is entirely possible that *hgcAB* expression may indeed not be connected to actual methylmercury production, future research addressing these questions will overall expand our currently limited understanding of *hgcAB* expression.

### Conclusions.

In this study, we expanded upon the known diversity of microorganisms that are likely to perform mercury methylation. We demonstrated that putative methylators encompass 30 phylum-level lineages and diverse metabolic guilds using a set of publicly available isolate genomes, MAGs, and novel freshwater MAGs. Apparent extensive HGT of the diverse *hgcAB* region poses unique challenges for detecting methylating populations in the environment, in which “universal” amplicon or even group-specific approaches may not accurately reflect the true phylogenetic origin of specific methylators. Furthermore, genome-resolved metatranscriptomics of putative methylators in a thawing permafrost system revealed that specific methylating populations are transcriptionally active at different sites and depths. These results highlight the importance of moving beyond identifying the mere presence of a gene to infer a specific function and investigating transcriptionally active populations. Overall, genome-resolved omics techniques are an appealing approach for accurately assessing the controls and constraints of microbial MeHg production in the environment.

## MATERIALS AND METHODS

### Accessed data sets, sampled sites, and metagenomic assembly.

We used a combination of publicly available MAGs, sequenced isolates, and newly assembled MAGs from three freshwater lakes. We sampled Lake Tanganyika in the East African Rift Valley, Lake Mendota in Madison, WI, and Trout Bog Lake near Minocqua, WI. We collected depth-discrete samples along the northern basin of Lake Tanganyika at two stations (Kigoma and Mahale). DNA extraction, metagenomic sequencing of 24 samples from Lake Tanganyika, and subsequent genome assembly and refinement were performed as described by Tran et al. (34). Briefly, 24 samples were passed through a 0.2-μm-pore-size fraction filter and used for shotgun metagenomic sequencing on the Illumina HiSeq 2500 platform at the Department of Energy Joint Genome Institute. Each of the 24 metagenomes was individually assembled with metaSPAdes and binned into population genomes using a combination of MetaBat, MetaBat2, and MaxBin (81–83). Across these binning approaches, approximately 4,000 individual bins were assembled. To dereplicate identical bins assembled through different platforms and from different samples, we applied DasTool and dRep to keep the highest-quality bin for a given cluster, resulting in 803 total MAGs (84, 85). Using completeness and contamination cutoffs of >70% and <10%, respectively, this resulted in 431 MAGs. From this data set, we used 16 MAGs that we identified as confident *hgcA* hits from criteria described below.

Five depth-discrete samples were collected from the Lake Mendota hypolimnion using a peristaltic pump onto a 0.2-μm Whatman filter and were used for Illumina shotgun sequencing on the HiSeq 4000 at the QB3 sequencing center in Berkeley, CA, as described by Peterson et al. (33). Samples were individually assembled using metaSPAdes, and population genomes were constructed using a combination of the MetaBat2, Maxbin, and CONCOCT binning algorithms (81–83, 86). Resulting MAGs were aggregated with DasTool and dereplicated into representative sets by pairwise average nucleotide identity comparisons (84). This resulted in a total of 228 bins that were each manually refined and inspected for uniform differential coverage using anvi’o (87), 35 of which were used in this study through identifying confident *hgcA* hits from the criteria described below.

Population genomes from samples collected from Trout Bog Lake were assembled and manually curated as previously described (35, 88, 89), from which we used four genomes that contained confident *hgcA* hits from the criteria described below. All publicly available isolate genomes and MAGs were accessed from GenBank in August of 2019, resulting in >200,000 genomes in which to search for *hgcA.* Genomes listed by Jones et al. (29) were accessed from JGI/IMG in March of 2019 at GOLD study identification (ID) Gs0130353. Only genomes of medium quality according to MiMAG standards with >50% completeness and <10% redundancy were kept for downstream analyses, as calculated with CheckM (90, 91).

### Identification of putative methylators.

We built a hidden Markov model (HMM) profile of the HgcA protein using a collection of full-length HgcA protein sequences from experimentally verified methylating organisms (19). The constructed HMM profile was then used to identify putative mercury-methylating bacteria and archaea. For all genome sequences, open reading frames and protein-coding genes were predicted using Prodigal (92). We used hmmsearch in the hmmer program to search all predicted protein sequences with the HMM profile with an E value cutoff of 1e−50 (93). Sequence hits with an E value cutoff score <1e−50 and/or a score of 300 were removed from the results to ensure high confidence in all hits. All archaeal and bacterial HgcA hits were concatenated and aligned with MAFFT (94). Alignments were manually visualized using AliView (95), and sequences without the conserved cap-helix domain [G(I/V)NVWCAAGK] reported by Parks et al. (19) were removed. Although these cutoff criteria may be stringent, we found that an E value cutoff of 1e−50 and a threshold cutoff score of 300 returned hits that nearly all contained the conserved cap-helix domain and manual inspection and curation of the resulting alignment were further reduced.

From this set of putative methylators, the taxonomy of each genome was assigned and/or confirmed using both automatic and manual classification approaches. Each genome was automatically classified using the genome taxonomy database toolkit (GTDB-tk) with default parameters (52). Additionally, each genome was manually classified using a set of 16 ribosomal protein markers (96). If a genome’s taxonomy could not be resolved between the GTDB-tk classification and the manual ribosomal protein classification method, the genome was removed from the data set (approximately 30 genomes). We kept specific genomes designated “unclassified,” as a few genomes with this designation belong to newly assigned phyla that have not been characterized in the literature, such as the proposed phyla BMS3A and Moduliflexota in the GTDB. This resulted in a total of 904 putative methylators used for downstream analyses as described. All genome information for each putative methylator, including taxonomic assignment by each method, quality, and genome statistics, is provided in Table S1 available at https://figshare.com/articles/dataset/mehg-metadata/10062413 and summarized in Fig. S1 in the supplemental material.

### *hgcAB* and ribosomal phylogenies.

We selected the highest-quality methylator genome from each phylum (total of 30 individual phyla containing a putative methylator) to place in a prokaryotic tree of life. Bacterial and archaeal references (the majority from Anantharaman et al. [39]) were screened for the 16 ribosomal protein HMMs along with the select 30 methylators. Individual ribosomal protein hits were aligned with MAFFT (94) and concatenated. The tree was constructed using FastTree and further visualized and edited using the interactive tree of life (iTOL) online tool (97, 98) to highlight methylating phyla and novel methylating groups.

We created an HMM profile of the HgcB protein using full-length HgcB sequences from experimentally verified methylators as described above for HgcA (17, 93). We searched for HgcB in the set of 904 putative methylators identified from HgcA presence as described above. We were able to identify HgcB-like sequences in all 904 putative methylators but further screened these hits as follows. All putative HgcB hits had to have a threshold cutoff score greater than 70 and contain the conserved ferredoxin-binding motif (CXXCXXXC) as reported by Parks et al. (19). Additionally, we required the resulting hit to be either directly downstream of *hgcA* or within five open reading frames, as some experimentally verified methylators, such as Desulfovibrio africanus Walvis Bay, have been observed to have genes between *hgcAB* (19). The resulting HgcB hit also had to be on the same contig as HgcA to ensure a confident hit and potentially include it in the HgcAB phylogeny. We recognize that this criterion might be overly conservative and stringent, potentially excluding some genomes with both genes (e.g., *hgcB* on a separate contig) or fused *hgcAB* hits, but we preferred to avoid any false positives. This resulted in 844 of 904 identified methylators containing a confident HgcB hit to be used for the HgcAB phylogeny. A given genome had to contain 12 or more ribosomal protein markers to be included in the HgcAB tree used to compare against a concatenated ribosomal protein phylogeny. Additionally, we removed sequences with low bootstrap support using RogueNaRok (99). Overall, this resulted in 650 HgcAB sequences used to create a representative HgcAB tree to compare to the corresponding concatenated ribosomal protein tree.

The HgcAB protein sequences were aligned separately using MUSCLE and concatenated (100). The alignment was uploaded to the Galaxy server for filtering with BMGE1.1 using the BLOSUM30 matrix, a threshold and gap rate cutoff score of 0.5, and a minimum block size of 5 (101, 102). Four fused HgcAB sequences were obtained from GenBank for Kosmotoga pacifica (WP_047755538), Methanococcoides methylutens (WP_048193041), Pyrococcus furiosus DSM 3638 (AAL80853), and an unclassified *Atribacteria* (WP_018206138) as used by Gionfriddo et al. (27) as paralogs to root the tree and aligned using MUSCLE (100). A consensus alignment of the concatenated HgcAB sequences and the fused HgcAB sequences was created with MUSCLE (100). A phylogenetic tree of the hits was constructed using RAxML with 100 rapid bootstraps (103). The tree was rooted with the four fused HgcAB sequences and edited using iTOL (98). For visualization ease, we collapsed clades by the dominant monophyletic phylum when possible. For example, if a monophyletic clade contained a majority (approximately 80% to 90%) of Deltaproteobacteria sequences and one *Nitrospirae* sequence, we collapsed and identified the clade as a whole as Deltaproteobacteria. This was performed to highlight broad trends in the disparate phylogenetic structure of identified HgcAB sequences. To reduce complexity, identified HgcAB sequences from candidate phyla containing few individuals were not assigned individual colors.

The corresponding ribosomal tree derived from genomes carrying the representative HgcAB sequences was constructed using a collection of 16 ribosomal proteins provided in the metabolisHMM package (96, 104), requiring that each putative methylator contain at least 12 ribosomal proteins. Each marker was searched for in each genome using specific HMM profiles for each ribosomal protein, individually aligned using MAFFT (94), and then concatenated together. A phylogenetic tree was constructed using RAxML with 100 bootstraps and visualized using iTOL (98, 103). A phylogenetic tree of HgcAB with noncollapsed branches, most resolved taxonomical names, and bootstrap scores is provided in Fig. S4 available at https://figshare.com/articles/figure/hgcAB_annotated_tree_full/12592085.

### Functional annotations, metabolic reconstructions, and *in silico* PCR analysis.

All genomes were functionally annotated using Prokka (105). The predicted gene sequence for each *hgcA* open reading frame was obtained using the locus tag of the predicted *hgcA* protein with the above-described HMM-based approach. *In silico* PCR with the broad-range *hgcA* primers and group-specific qPCR *hgcA* primers were tested using Geneious (Biomatters Ltd., Auckland, NZ), with primer sequences from Christensen et al. (22). The settings allow for both 0 and 2 mismatches, and the primer amplification “hit” results were compared to the full-genome classification of the given *hgcA* gene sequence. For genome synteny analysis of select permafrost methylators, Easyfig was used to generate pairwise BLAST results and view alignments (106). We did not use one of the *Myxococcales* MAGs as it was exactly identical to the other. The *Opitutae* genome GCA_003154355 from the UBA assembled set was not used, because *hgcA* was found on only a very short contig.

Broad metabolic capabilities were characterized across all methylating organisms based on curated sets of metabolic HMM profile sets provided in the metabolisHMM package (39, 104). An HMM of the putative transcriptional regulator was built using the alignment of the five putative transcriptional regulators with similar *hgcAB* sequences with MUSCLE and hmmbuild (93, 100) and searched for among all methylators with hmmsearch (93). A genome was considered to have a hit for the putative transcriptional regulator if the identified protein contained a search hit E value of greater than 1e−10 and was immediately upstream of *hgcAB* or 1 to 2 genes upstream. Raw presence/absence results are provided in Table S5 at https://figshare.com/articles/dataset/raw-metabolic-marker-results-high-qual-genomes/10203530.

Metabolic reconstruction of the MENDH-*Thermoleophilia* bin was supplemented with annotations using the KofamKOALA distribution of the KEGG database and parsed for significant hits (107) (annotations available in Table S2 at https://figshare.com/articles/dataset/MENDH-Thermo-annotations/11620104). The phylogeny of the MENDH-*Thermoleophilia* MAG was confirmed by downloading representative/reference sequences from GenBank and Refseq for the entire *Actinobacteria* phylum, depending on which database/designation was available for the particular class. All *Thermoleophilia* class genomes were downloaded as designated by the Genome Taxonomy Database (52). We included Bacillus subtilis strain 168 as an outgroup. The phylogeny was constructed using the alignment of concatenated ribosomal proteins as described above. The tree was constructed using RAxML with 100 rapid bootstraps and visualized with iTOL (98, 103).

### Transcriptional activity of putative methylators in a permafrost system.

To investigate the transcriptional activity of methylators in the environment, we tracked transcriptional activity of the identified methylating organisms in a permafrost thaw gradient. (31). All 26 raw metatranscriptomes from the palsa, bog, and fen sites sequenced on the NextSeq Illumina platform were downloaded from NCBI from BioProject accession number PRJNA386568. Paired-end sequences were quality filtered and adapters removed using fastp (108). Each transcriptome was mapped to the indexed open reading frames of the 111 putative methylators identified in this ecosystem using kallisto (109). Annotated open reading frames and size in base pairs for each genome were supplied using Prokka (105). For every sample, counts were normalized by calculating transcripts per million (TPM) (110).

Open reading frames for *hgcA*, *hgcB*, and a putative conserved regulator were identified for each genome from the HMM profile annotations as described above. We did not detect expression of any of the putative methylators in any of the palsa samples and therefore did not include these samples in the results. Expression for *hgcA*, *hgcB*, and the regulator within a phylum was calculated by total TPM expression of that gene within that phylum. For *hgcA*, total *hgcA* expression within a sample was calculated as the total TPM-normalized expression of *hgcA* counts within that sample. The total average expression of methylators within a phylum was calculated by adding all TPM-normalized counts for all genomes within a phylum and averaged by the number of genomes within that phylum to represent the average activity of that group compared to *hgcA* activity. Expression of *hgcA* was compared to that of the housekeeping gene *rpoB*, which encodes the RNA polymerase beta subunit and predicted from Prokka annotations.

### Data availability.

Lake Tanganyika genomes are available at https://osf.io/pmhae/. Lake Mendota genomes are available at https://osf.io/9vwgt/. The four Trout Bog genomes can be accessed from JGI/IMG under accession IDs 2582580680, 2582580684, 2582580694, and 2593339183. A complete workflow including all code and analysis workflows can be found at https://github.com/elizabethmcd/MEHG. All supplementary data files and tables are available on Figshare at https://figshare.com/projects/Expanded_Diversity_and_Metabolic_Flexibility_of_Microbial_Mercury_Methylation/70361 under the CC-BY 4.0 license.
