# Supplementary figures and images for: Expanded Phylogenetic Diversity and Metabolic Flexibility of Mercury-Methylating Microorganisms
Source: mSystems. 2020 Aug 18;5(4):e00299-20. doi: 10.1128/mSystems.00299-20 (PMC7438021; doi:10.1128/mSystems.00299-20)

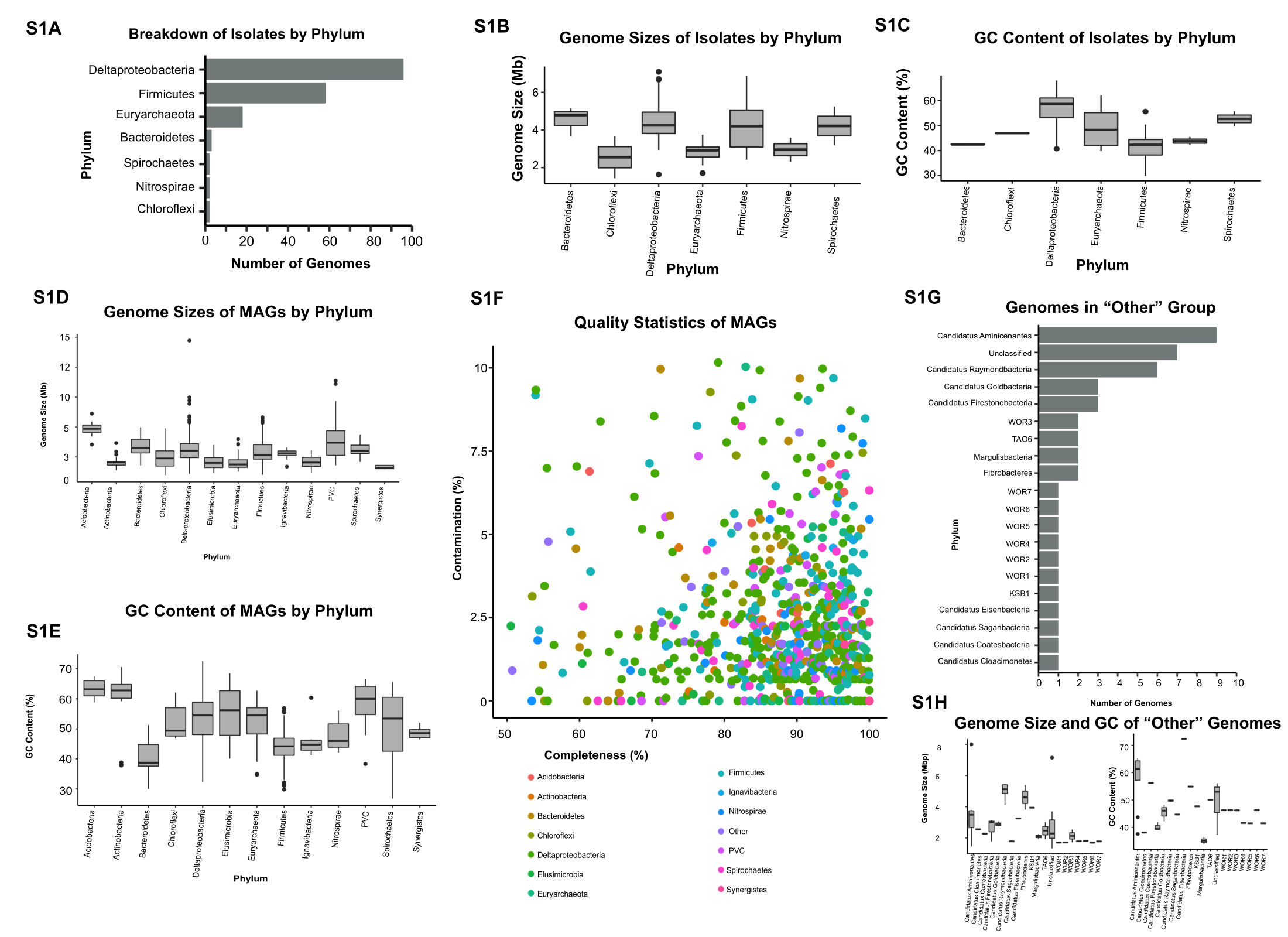

Supplement: FIG S1 [file mSystems.00299-20-sf001.tif]

S2A.

hgcB Expression

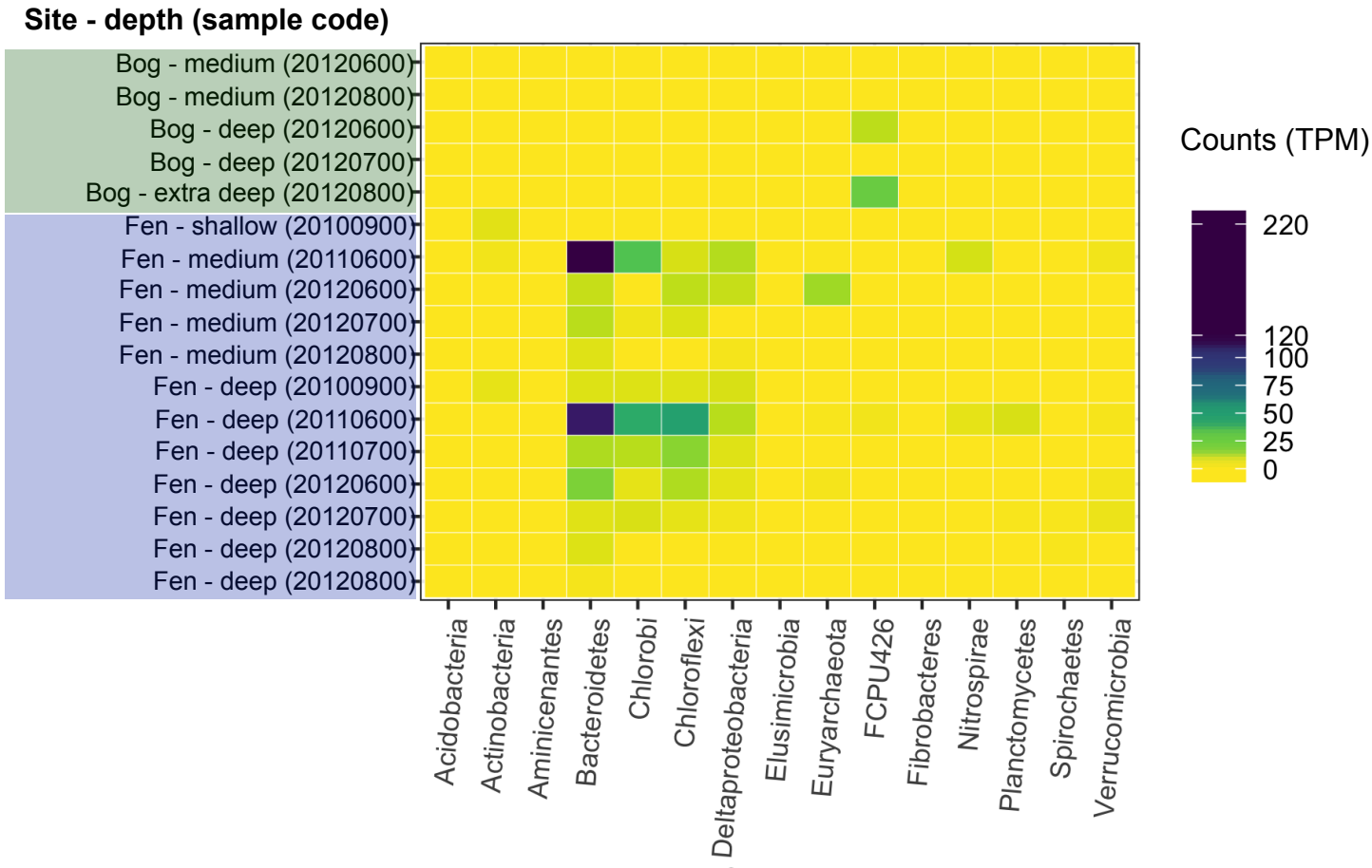

S2B.

Putative Regulator Expression

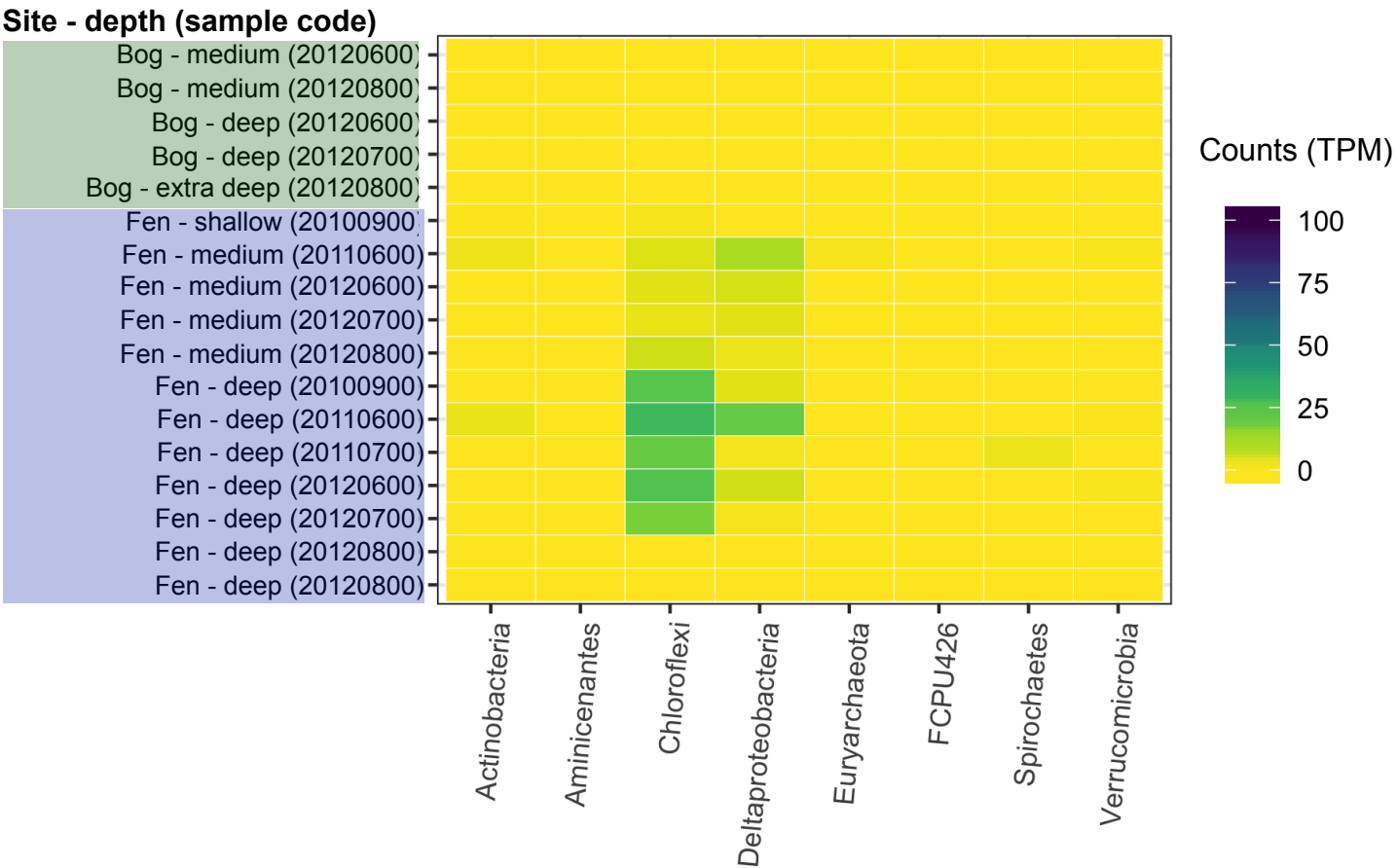

Supplement: FIG S2 [file mSystems.00299-20-sf002.pdf]
