# Supplementary material for: Expanded Phylogenetic Diversity and Metabolic Flexibility of Mercury-Methylating Microorganisms
Source: mSystems. 2020 Aug 18;5(4):e00299-20. doi: 10.1128/mSystems.00299-20 (PMC7438021; doi:10.1128/mSystems.00299-20)
Supplement: FIG S3 [file mSystems.00299-20-sf003.pdf]

S3.

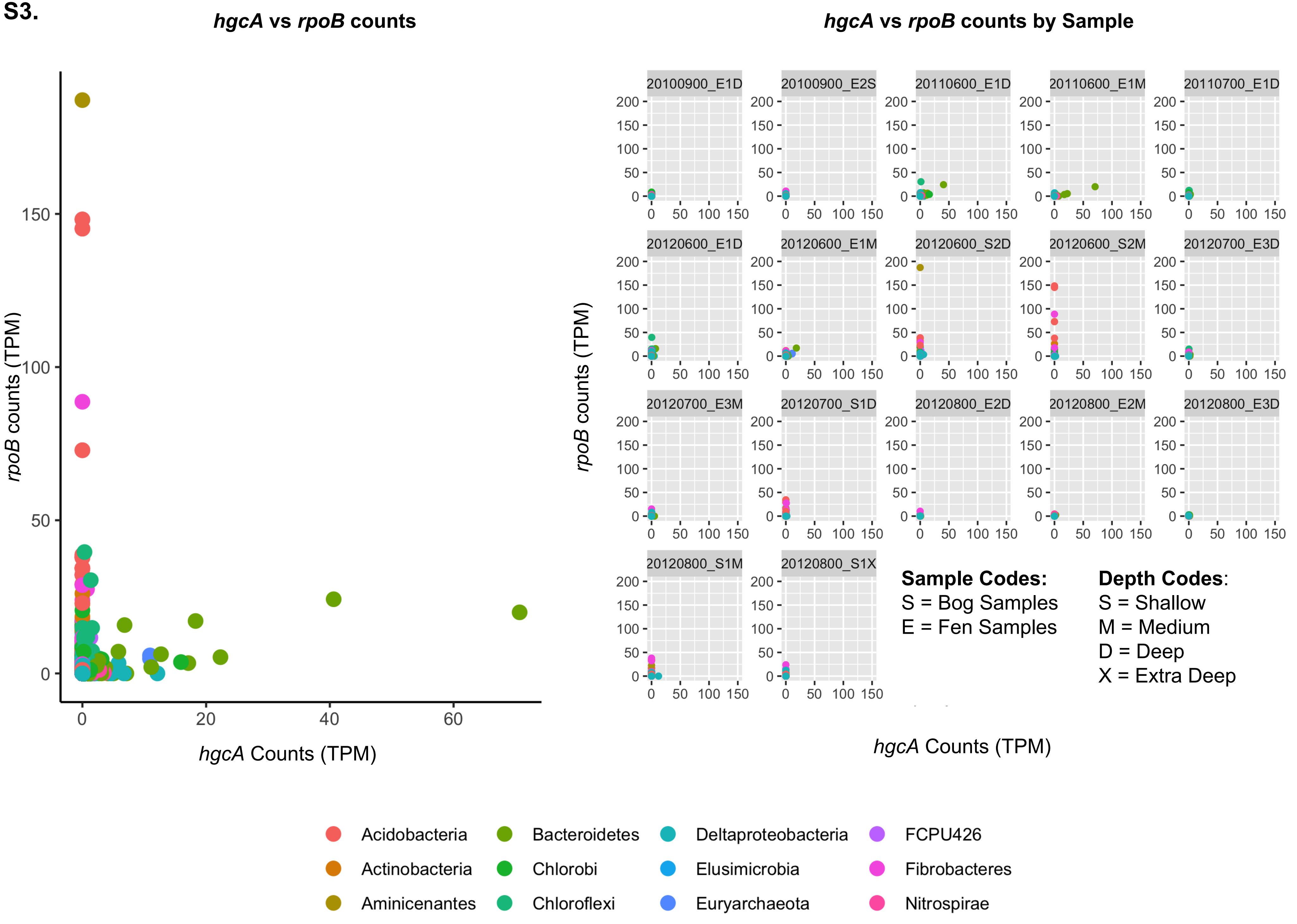

hgcA vs rpoB counts
rpoB counts (TPM)
hgcA Counts (TPM)
hgcA vs rpoB counts by Sample
20100900_E1D
20100900_E2S
20110600_E1D
20110600_E1M
20110700_E1D
20120600_E1D
20120600_E1M
20120600_S2D
20120600_S2M
20120700_E3D
20120700_E3M
20120700_S1D
20120800_E2D
20120800_E2M
20120800_E3D
20120800_S1M
20120800_S1X
Sample Codes:
S = Bog Samples
E = Fen Samples
Depth Codes:
S = Shallow
M = Medium
D = Deep
X = Extra Deep
Acidobacteria
Actinobacteria
Aminicenantes
Bacteroidetes
Chlorobi
Chloroflexi
Deltaproteobacteria
Elusimicrobia
Euryarchaeota
FCPU426
Fibrobacteres
Nitrospirae
